# Interplay of Epigenetic Reprogramming, Mitochondrial Metabolism, and Dopamine Signalling Pathways Uncovers Metabolic Vulnerabilities in Diffuse Midline Glioma

**DOI:** 10.3390/cancers18142186

**Published:** 2026-07-08

**Authors:** Han Shen, Yizhou Huang, Kristina M. Cook, Eric Hau

**Affiliations:** 1 Translational Radiation Biology and Oncology Group, Centre for Cancer Research, The Westmead Institute for Medical Research, Westmead, NSW 2145, Australia; kristina.cook@sydney.edu.au (K.M.C.); eric.hau@health.nsw.gov.au (E.H.); 2 Faculty of Medicine and Health, The University of Sydney, Camperdown, NSW 2050, Australia; 3 Functional Genomics of Leukaemia, Children’s Cancer Institute at Minderoo Children’s Comprehensive Cancer Centre, Randwick, NSW 2031, Australia; jhuang@ccia.org.au; 4 School of Clinical Medicine, Faculty of Medicine, The University of New South Wales Sydney, Kensington, NSW 2052, Australia; 5 The Western Sydney Radiation Oncology Network, Sydney, NSW 2145, Australia

**Keywords:** DMG, mitochondria, epigenetics, metabolism, dopamine

## Abstract

Diffuse midline glioma (DMG) is one of the most aggressive paediatric brain tumours and remains almost universally fatal despite decades of research. The defining molecular feature of approximately 80% of DMG tumours is H3K27M, which disrupts PRC2 activity and profoundly remodels chromatin architecture. Increasing evidence suggests that this epigenetic alteration not only rewires transcriptional programs but also influences tumour metabolism. Several studies indicate that H3K27M-mutant tumours exhibit altered mitochondrial metabolism, oxidative phosphorylation activity, redox regulation, and cellular stress responses, although the extent of oxidative phosphorylation dependence varies between models, tumour subtypes, and cellular states. In parallel, dopaminergic signalling has been implicated in cancer stem cell maintenance, metabolic regulation, and tumour survival across multiple malignancies, including glioma. The imipridone compound ONC201/dordaviprone, initially described as a dopamine receptor D2/3 antagonist and subsequently characterised as a mitochondrial ClpP agonist, demonstrates clinical activity in H3K27M-mutant DMG and induces mitochondrial stress responses. In this review, we examine emerging connections between epigenetic dysregulation, mitochondrial metabolism, and dopamine signalling in DMG. We propose that H3K27M-driven epigenetic reprogramming may impose metabolic constraints that increase tumour reliance on mitochondrial bioenergetics and stress-buffering pathways. Within this context, dopamine signalling may function as a metabolic rheostat that contributes to mitochondrial homeostasis; however, this remains a hypothesis requiring direct experimental validation in DMG models. Pharmacologic disruption of this axis may destabilise tumour metabolism and expose therapeutically exploitable vulnerabilities in this otherwise treatment-resistant disease.

## Simple Summary

Diffuse midline glioma is an aggressive childhood brain tumour for which radiotherapy remains the main treatment, but durable control is uncommon. Many tumours carry the H3K27M alteration, which changes chromatin regulation and may also constrain tumour metabolism. This review explains how H3K27M-driven epigenetic reprogramming may increase reliance on mitochondrial metabolism and cellular stress-buffering pathways. We also discuss dopamine receptor signalling and the clinical development of imipridones, such as ONC201/dordaviprone, which connect dopamine receptor biology with mitochondrial stress responses. Because the proposed dopamine–mitochondria rheostat remains a conceptual model, we distinguish established evidence from hypotheses requiring further validation. Understanding these pathways may support more rational combination strategies for this treatment-resistant disease.

## 1. Introduction

Diffuse midline glioma (DMG) represents one of the most devastating paediatric central nervous system malignancies. Previously classified as diffuse intrinsic pontine glioma (DIPG) when arising in the pons, these tumours occur within midline structures, including the pons, thalamus, and spinal cord. Their highly infiltrative growth pattern and critical anatomical location make surgical resection impossible in most cases. Although chemotherapy, targeted therapies, immunotherapy, and re-irradiation strategies have been explored, radiotherapy remains the backbone of initial treatment, and median survival remains approximately 9–12 months [1,2]. Recent reviews have highlighted the evolving clinical and biological understanding of H3K27M-mutant DIPG/DMG, including advances in biopsy, radiotherapy, re-irradiation, systemic and targeted therapies, immunotherapy, tumour–microenvironment interactions, and emerging metabolic vulnerabilities [3,4,5,6]. Despite these advances, durable disease control remains uncommon, underscoring the need to better define tumour-intrinsic vulnerabilities that arise from the defining epigenetic and metabolic features of this disease.

The discovery of recurrent histone mutations has fundamentally transformed the understanding of DMG biology. Approximately 80% of tumours harbour a lysine-to-methionine substitution at position 27 of histone H3 (H3K27M), occurring in either the H3.3 variant encoded by *H3F3A* or the H3.1 variant encoded by *HIST1H3B/C* [7,8]. This mutation interferes with the catalytic activity of Polycomb Repressive Complex 2 (PRC2), leading to global reduction in the repressive histone mark H3K27me3 [9]. Consequently, the chromatin landscape becomes extensively reprogrammed, allowing aberrant activation of developmental gene expression programs that contribute to tumourigenesis [10,11].

While the epigenetic consequences of the H3K27M mutation have been studied extensively, emerging work suggests that this profound chromatin dysregulation may act as a primary driver of metabolic rewiring. Cancer metabolism and epigenetic regulation are deeply interconnected. Just as chromatin-modifying enzymes depend on mitochondrial metabolites like α-ketoglutarate, acetyl-CoA, and S-adenosylmethionine [12,13], primary epigenetic alterations can fundamentally dictate cellular metabolic states. In the context of DMG, the widespread transcriptional shifts resulting from H3K27M-mediated reprogramming enforce changes in metabolic flux, compelling the tumour cells to alter their metabolic pathways to sustain their altered chromatin landscape. For example, integrated metabolic–epigenomic studies show enhanced glycolysis, glutaminolysis, TCA cycle activity and alpha-ketoglutarate production in H3K27M DIPG, while related DMG/pHGG models identify mitochondrial bioenergetic, hypoxia-associated and differentiation-state-dependent metabolic vulnerabilities [14,15,16]. Consistent with this epigenetic-driven metabolic shift, recent studies integrating transcriptomic and epigenomic profiling have revealed coordinated changes in metabolic gene expression programs in DMG [17,18]. These studies suggest that metabolic rewiring may represent an important component of DMG tumour biology. Additionally, several paediatric high-grade glioma models demonstrate enrichment of mitochondrial oxidative phosphorylation (OXPHOS) pathways, suggesting that mitochondrial metabolism may represent a key vulnerability in these tumours [14,19,20].

At the same time, growing evidence indicates that neuronal signalling pathways can regulate glioma growth. Activity-dependent secretion of neuroligin-3 and synaptic interactions between neurons and tumour cells promote proliferation and tumour progression in glioma [21]. Subsequent work demonstrated activity-regulated NLGN3 dependency and direct glutamatergic neuron–glioma synapses that drive glioma depolarisation, proliferation and circuit integration [22,23,24]. While glutamatergic signalling has received substantial attention, dopaminergic pathways are also present within the brain microenvironment and may influence tumour biology. Dopamine receptor signalling has been implicated in cancer stem cell regulation, tumour metabolism, and therapeutic resistance in several malignancies [25,26,27]. In glioma, dopamine receptor D2 (DRD2) expression has been associated with tumour-initiating cell populations and metabolic adaptations that support tumour growth [26]. Importantly, pharmacologic antagonism of dopamine receptors using imipridone compounds such as ONC201 has demonstrated encouraging clinical activity in patients with H3K27M-mutant DMG [28,29,30].

In this review, we examine how epigenetic reprogramming driven by the H3K27M mutation may interact with metabolic pathways and mitochondrial function in DMG. We then discuss emerging evidence linking dopamine signalling to tumour metabolism and propose a conceptual framework in which dopaminergic pathways may contribute to stabilising mitochondrial homeostasis in epigenetically constrained tumour cells. We explicitly distinguish established evidence from proposed mechanisms to clarify the current limits of this model.

## 2. Epigenetic Reprogramming and Metabolic Constraint in DMG

The H3K27M mutation exerts its oncogenic effects primarily through disruption of PRC2-mediated chromatin repression. By binding to the catalytic subunit EZH2 of PRC2, the mutant histone inhibits deposition of H3K27 trimethylation (H3K27me3) across the genome [9]. This results in widespread loss of repressive chromatin domains and activation of transcriptional programs that are normally tightly regulated during neural development [18,31]. Global reduction in H3K27me3 also leads to altered chromatin accessibility across numerous regulatory elements, resulting in deregulation of developmental pathways controlling neural progenitor proliferation, differentiation, and lineage commitment [10,11].

A growing body of evidence indicates that chromatin regulation and cellular metabolism are closely interconnected processes. In cancer, epigenetic modifications are now recognised as primary determinants of metabolic architecture [32]. For example, in gastric, colon, and breast cancers, promoter hypermethylation frequently silences fructose-1,6-bisphosphatase isoforms (FBP-1/2) [33,34,35]. By antagonising gluconeogenesis, this epigenetic lock enforces a highly glycolytic phenotype to sustain rapid biomass production [34,35]. Similarly, promoter hypomethylation upregulates hexokinase 2 (HK2) in hepatocellular carcinoma, accelerating glucose sequestration to fuel glycolytic flux [36]. In H3K27M-mutant DMG, the primary epigenetic alterations also actively dictate and constrain a cell’s metabolic state. By reshaping transcriptional programs and locking cells into specific developmental lineage states, the H3K27M-driven epigenetic reprogramming coerces the tumour into rewiring its metabolic flux [37]. This top-down epigenetic regulation forces the cell to restructure its metabolic networks, often increasing reliance on mitochondrial metabolism, to ensure a continuous supply of the energetic and biosynthetic substrates necessary to sustain its oncogenic chromatin landscape and support rapid proliferation [15].

Moreover, this metabolic–epigenetic coupling operates as a bidirectional loop and can reinforce oncogenic transcriptional networks and support tumour progression [38]. In the context of H3K27M DMG, where chromatin regulation is already profoundly altered, metabolic perturbations may further modulate the epigenetic landscape and influence tumour cell behaviour. Mitochondrial metabolism is particularly important in this context because the tricarboxylic acid (TCA) cycle produces several metabolites that serve as substrates or regulators of chromatin-modifying enzymes. For example, citrate exported from the mitochondria can be converted into acetyl-CoA in the cytosol and nucleus, thereby providing a substrate for histone acetylation reactions [39]. Similarly, α-ketoglutarate generated through the TCA cycle supports the activity of histone 3 demethylases that regulate chromatin structure [40]. In H3K27M DIPG, Chung et al. demonstrated that enhanced glucose and glutamine metabolism increases alpha-ketoglutarate, which helps maintain global H3K27 hypomethylation through KDM6A/B-dependent demethylation; in contrast, mutant IDH1/2 consumes alpha-ketoglutarate to generate D-2-hydroxyglutarate, inhibits alpha-ketoglutarate-dependent demethylases, increases H3K27me3, and is mutually exclusive/synthetically lethal with H3K27M [15,41].

Taken together, these observations suggest that H3K27M-driven epigenetic reprogramming may impose constraints on tumour metabolism. By reshaping transcriptional programs and developmental lineage states, the mutation may limit the range of metabolic configurations available to tumour cells. Such metabolic constraint could increase reliance on specific pathways, particularly mitochondrial OXPHOS and associated stress-buffering systems. If this model is correct, metabolic dependencies created by epigenetic reprogramming may represent important therapeutic vulnerabilities in DMG.

## 3. Mitochondrial Metabolism as a Therapeutic Vulnerability in DMG

### 3.1. Mitochondrial Metabolism as a Central Metabolic Hub

Mitochondria play a central role in cellular metabolism, integrating bioenergetic, biosynthetic, and signalling functions [42,43]. In addition to producing ATP through OXPHOS, mitochondria regulate the generation of metabolic intermediates required for macromolecule synthesis, control cellular redox balance, and coordinate stress-response pathways [42,43]. Through the TCA cycle, mitochondrial metabolism provides critical substrates for nucleotide, lipid, and amino acid biosynthesis, thereby supporting the anabolic demands of rapidly proliferating tumour cells [44]. Mitochondria also participate in calcium homeostasis, apoptosis regulation, and epigenetic control through the generation of metabolites such as acetyl-CoA, α-ketoglutarate, succinate, fumarate, and S-adenosylmethionine, all of which influence chromatin-modifying enzymes and transcriptional programs [45,46,47]. Because of these diverse functions, mitochondrial metabolism represents a major determinant of tumour cell survival, proliferation, and therapeutic resistance.

Historically, cancer metabolism has often been discussed in the context of the Warburg effect, in which tumour cells preferentially utilise glycolysis even in the presence of oxygen [48]. This metabolic shift was initially interpreted as evidence that mitochondrial respiration was dispensable in cancer cells. However, it is now widely recognised that mitochondrial respiration remains essential for many malignancies [49]. Although glycolysis provides rapid ATP generation and facilitates adaptation to hypoxic conditions, OXPHOS offers substantially greater energetic efficiency and supports anabolic pathways required for tumour growth. Mitochondrial respiration is also essential for maintaining redox balance and regenerating metabolites required for biosynthetic reactions. Consequently, many cancers exhibit metabolic plasticity, dynamically switching between glycolysis and mitochondrial respiration depending on nutrient availability, oxygen tension, oncogenic signalling, and therapeutic stress.

### 3.2. Mitochondrial Dependency in Paediatric High-Grade Gliomas and DMG

Emerging evidence highlights the critical role of mitochondrial metabolism in paediatric brain tumours. Several studies suggest that paediatric high-grade gliomas display metabolic characteristics distinct from those observed in adult glioblastoma. Whereas adult glioblastomas frequently exhibit strongly glycolytic phenotypes, paediatric tumours appear to retain substantial reliance on mitochondrial oxidative metabolism [14,19,50]. This distinction may partly reflect the developmental context and cellular origins of paediatric gliomas, which arise from neural precursor populations with inherently different metabolic states compared with adult tumours [18,51,52,53]. In addition, the epigenetic dysregulation imposed by H3K27M mutations may constrain metabolic lineage programs and favour mitochondrial dependency. Consistent with this concept, metabolomic and transcriptomic profiling studies have demonstrated enrichment of OXPHOS-associated pathways in several paediatric high-grade gliomas, including DMG [14,19]. H3K27M-mutant DMG tumours exhibit elevated expression of genes associated with electron transport chain activity, mitochondrial protein translation, and mitochondrial biogenesis [17,18]. Furthermore, patient-derived DMG models demonstrate heightened sensitivity to pharmacological disruption of mitochondrial respiration, supporting the concept that mitochondrial metabolism represents a functional vulnerability in this disease [14,16].

### 3.3. Oxidative Stress and Redox Homeostasis in DMG

Mitochondrial respiration inevitably generates reactive oxygen species (ROS) as by-products of electron transport chain activity, particularly at complexes I and III. Under physiological conditions, low levels of ROS function as signalling molecules that regulate proliferation, differentiation, and cellular adaptation. However, excessive ROS accumulation can lead to oxidative damage of DNA, proteins, and lipids, ultimately triggering apoptosis or other forms of cell death. Cancer cells therefore maintain a delicate balance between ROS production and antioxidant defence mechanisms. To cope with oxidative stress, tumour cells rely on antioxidant systems, including glutathione metabolism, thioredoxin pathways, superoxide dismutases, catalase, and NADPH-dependent redox buffering systems. NADPH generated through the pentose phosphate pathway, malic enzyme activity, and mitochondrial one-carbon metabolism is particularly important for sustaining antioxidant capacity.

DMG cells appear to exist close to the threshold of tolerable oxidative stress due to their high mitochondrial activity [19,54]. As a consequence, additional perturbation of mitochondrial respiration can drive catastrophic ROS accumulation and oxidative damage. Several studies have demonstrated that therapies disrupting mitochondrial function induce profound oxidative stress in DMG models, suggesting that these tumours may possess limited metabolic reserve capacity [14,16,19]. This concept has important therapeutic implications because agents that either increase ROS production or impair antioxidant defences may selectively target tumour cells while sparing normal tissues with greater redox flexibility.

### 3.4. Integrated Stress Response and Mitochondrial Stress Adaptation

In addition to regulating redox balance, mitochondria serve as critical sensors of cellular stress. Perturbations in mitochondrial function activate signalling pathways that coordinate adaptive responses aimed at restoring metabolic homeostasis. One of the most important adaptive pathways is the integrated stress response (ISR). ISR activation occurs when stress-sensing kinases, including PERK, GCN2, PKR, and HRI, phosphorylate the translation initiation factor eIF2α [55]. These kinases respond to distinct stress signals, including endoplasmic reticulum stress, amino acid deprivation, viral infection, heme deficiency, mitochondrial dysfunction, and oxidative stress. Phosphorylation of eIF2α suppresses global protein translation while selectively enhancing translation of stress-responsive transcription factors, such as activating transcription factor 4 (ATF4) [55,56].

ATF4 orchestrates transcriptional programs that promote adaptation to cellular stress [57]. These programs regulate amino acid transport and biosynthesis, glutathione metabolism, autophagy, mitochondrial recovery, and antioxidant defence pathways [55]. Through these mechanisms, the ISR enables cells to survive transient metabolic or proteotoxic stress. However, prolonged or excessive ISR activation can shift signalling toward apoptosis through induction of pro-apoptotic mediators, such as CHOP, DR5, and BIM [58]. This dual role positions the ISR as both a survival mechanism and a potential mediator of tumour cell death, depending on the magnitude and duration of stress exposure.

Recent evidence suggests that mitochondrial dysfunction also activates a more specific mitochondrial stress pathway termed the mitochondrial unfolded protein response (UPRmt) [59]. The UPRmt is triggered when mitochondrial proteostasis is disrupted by accumulation of unfolded or damaged proteins within the mitochondrial matrix [60]. Activation of this pathway induces transcriptional programs that enhance mitochondrial chaperone expression, protease activity, and metabolic adaptation [61]. Although the role of the UPRmt in DMG remains incompletely defined, emerging studies indicate that mitochondrial quality-control pathways may contribute to therapeutic resistance and tumour cell survival under metabolic stress [62]. Interactions between the ISR and UPRmt may therefore represent additional therapeutic vulnerabilities in DMG.

### 3.5. Therapeutic Targeting of Mitochondrial Vulnerabilities in DMG

Recent studies suggest that pharmacological disruption of mitochondrial function can effectively exploit these metabolic vulnerabilities in cancer cells. Historically, arsenic trioxide, a mitochondrial inhibitor and ROS inducer, has been successfully utilised in the treatment of acute promyelocytic leukaemia [63]. More recently, extensive preclinical studies have repurposed biguanides, demonstrating that both metformin and phenformin inhibit mitochondrial complex I activity, suppress ATP production, increase energetic stress, and extend survival in murine models of DMG [14,16]. Phenformin generally exhibits greater potency than metformin due to improved cellular uptake and stronger mitochondrial inhibition, although concerns regarding systemic toxicity have limited its clinical development.

Dichloroacetate (DCA), a pyruvate dehydrogenase kinase inhibitor, targets mitochondrial metabolism through a distinct mechanism. Rather than suppressing mitochondrial activity, DCA promotes pyruvate entry into the TCA cycle by activating pyruvate dehydrogenase, thereby shifting cellular metabolism from glycolysis toward mitochondrial OXPHOS [64]. In tumours already operating near maximal oxidative capacity, this forced mitochondrial activation can result in excessive ROS production, fatal redox imbalance, and cell death [64]. DCA has demonstrated promising preclinical activity both as a single agent and as a sensitiser to radiotherapy and chemotherapy [19,65,66]. Because radiation therapy itself induces ROS-mediated DNA damage, combination approaches that further enhance oxidative stress may prove particularly effective in DMG.

Imipridones are discussed in detail below because they sit at the intersection of dopamine receptor pharmacology and mitochondrial stress biology. From a metabolic perspective, ONC201/dordaviprone and related compounds activate the mitochondrial protease ClpP, promote degradation of respiratory chain and mitochondrial proteostasis proteins, suppress OXPHOS, and activate ISR programmes [58,59]. These observations support the broader concept that mitochondrial quality-control pathways can be therapeutically manipulated in DMG.

Additional therapeutic strategies targeting mitochondrial metabolism are also under investigation. Inhibitors of mitochondrial translation, mitochondrial chaperones, fatty acid oxidation, glutamine metabolism, and antioxidant systems have all demonstrated varying degrees of preclinical efficacy in glioma models [67]. Combination approaches may prove especially important because tumour cells frequently compensate for inhibition of one metabolic pathway by activating alternative nutrient utilisation programs [68,69]. Simultaneous targeting of mitochondrial metabolism and stress adaptation pathways may therefore enhance therapeutic efficacy and limit metabolic escape mechanisms.

### 3.6. Metabolic Heterogeneity and Plasticity in DMG

Importantly, the metabolic phenotype of DMG appears heterogeneous across studies, with some models showing strong OXPHOS signatures whereas others demonstrate increased glycolytic activity or mixed metabolic states [14,20,70]. These differences likely reflect several non-mutually exclusive variables: H3.1 versus H3.3 mutant background, developmental lineage state, anatomical site, regional oxygen and nutrient availability, prior therapy exposure, and differences between two-dimensional culture, spheroids, organoids, orthotopic xenografts, and patient specimens. Single-cell studies indicate that H3K27M gliomas contain oligodendrocyte progenitor-like, astrocytic-like, cycling, and more differentiated cellular states [18]. These states may differ in mitochondrial mass, redox buffering, glycolytic capacity, and stress-response dependence. Therefore, OXPHOS dependence should not be interpreted as a uniform property of all DMG cells but rather as a recurrent vulnerability enriched in particular molecular or cellular contexts.

Despite these complexities, multiple lines of evidence mentioned in the above sections indicate that mitochondrial metabolism remains central to tumour survival in many DMG models. Mitochondria integrate bioenergetic demands, redox regulation, stress adaptation, and epigenetic control, placing them at the centre of tumour cell fitness. However, therapeutic strategies should account for metabolic plasticity and the possibility that glycolytic subpopulations, oxidative subpopulations, and treatment-induced adaptive states may coexist within the same tumour. Combination approaches that jointly target mitochondrial metabolism, compensatory glycolysis, redox buffering, or adaptive stress signalling may therefore be more durable than single-pathway inhibition.

### 3.7. Comparative Glioma Context: Low-Grade Glioma and Adult Glioblastoma

Placing DMG within the broader glioma landscape helps clarify both shared and distinctive metabolic features. IDH-mutant lower-grade gliomas and secondary glioblastomas are dominated by neomorphic IDH1/2 activity and accumulation of D-2-hydroxyglutarate, an oncometabolite that inhibits alpha-ketoglutarate-dependent dioxygenases and drives widespread DNA and histone methylation changes [41]. In these tumours, metabolism and epigenetics are directly linked through mutant enzyme activity. By contrast, H3K27M-mutant DMG begins with a histone lesion that reshapes chromatin and developmental identity, with metabolic rewiring emerging downstream of this epigenetic constraint. Thus, both tumour classes demonstrate metabolic–epigenetic coupling, but the directionality and dominant initiating event differ.

Adult IDH-wild-type glioblastoma provides a second point of comparison. Adult glioblastoma is often described as highly glycolytic, yet glioma stem-like cells can retain substantial mitochondrial reserve capacity and can switch between glycolysis and OXPHOS under nutrient, oxygen, or therapeutic stress [26,50]. DRD2 signalling has been most clearly linked to metabolic plasticity in adult glioblastoma stem-like cells, where receptor activation promotes pseudohypoxic transcriptional programmes and supports tumour-propagating phenotypes [26]. DMG shares features of metabolic plasticity and neuronal microenvironmental dependence but differs by its paediatric developmental context, midline location, H3K27-altered chromatin state, and apparent sensitivity of subsets of models to mitochondrial stress. These comparisons support the rationale for studying dopamine–mitochondrial signalling in DMG while cautioning against extrapolating adult glioblastoma findings without direct validation in H3K27M-mutant models.

## 4. Dopamine Signalling and Antipsychotic Repurposing

### 4.1. Epidemiological Evidence Linking Dopamine Signalling to Cancer

Interest in dopamine receptor signalling in oncology initially emerged from epidemiologic observations. Several population-based studies have reported a reduced incidence of certain cancers among individuals treated for schizophrenia, raising the possibility that long-term exposure to antipsychotic medications may confer a protective effect [71,72]. Although these observations must be interpreted cautiously due to confounding factors such as lifestyle differences, smoking prevalence, and disparities in healthcare access, they nevertheless catalysed significant interest in the biological intersection between dopaminergic signalling and tumourigenesis. Subsequent experimental studies have supported the notion that dopamine signalling is not merely a neurophysiological process restricted to the central nervous system but also an important regulator of cancer cell behaviour across multiple tumour types [73].

### 4.2. Dopamine Receptor Biology and Oncogenic Signalling Pathways

At the cellular level, dopamine receptors have been shown to actively influence tumour growth, survival, and metabolic adaptation. Dopamine receptors are a family of G protein-coupled receptors broadly categorised into D1-like (DRD1 and DRD5) and D2-like (DRD2, DRD3, and DRD4) subtypes [74]. These receptor classes exert opposing effects on cyclic AMP signalling through differential coupling to stimulatory or inhibitory G proteins, thereby influencing a diverse range of downstream cellular processes [74]. In cancer cells, activation of dopamine receptors, particularly DRD2, regulates several critical oncogenic cascades, including the PI3K-AKT, MAPK-ERK, and mTOR pathways, which collectively govern cellular proliferation, bioenergetic metabolism, survival signalling, and stress adaptation [75]. Beyond these canonical pathways, DRD2 signalling has also been implicated in the regulation of hypoxia-inducible factors (HIFs), autophagic flux, and inflammatory transcriptional programs, suggesting a broader role in facilitating tumour plasticity under hostile microenvironmental conditions [76,77].

### 4.3. DRD2 Signalling in Glioma Stemness and Metabolic Plasticity

In glioma, DRD2 expression is significantly enriched in stem-like tumour cell populations, where it appears to promote metabolic states that favour tumour propagation and therapeutic resistance [26]. These glioma stem-like cells exhibit heightened metabolic flexibility and are capable of adapting to nutrient deprivation, oxidative stress, and hypoxic conditions encountered within the tumour microenvironment. Elegant mechanistic studies have demonstrated that DRD2 activation can induce a pseudohypoxic transcriptional programme characterised by stabilisation of HIF signalling even in normoxic conditions, thereby enhancing glycolysis, angiogenic signalling, and resistance to apoptosis [26]. Such findings are particularly relevant in DMG, where tumour progression is increasingly understood to depend on dynamic interactions between epigenetic dysregulation and metabolic rewiring. Because neuronal activity and neurotransmitter release are integral components of the brain microenvironment, dopamine may act not only as a systemic neurotransmitter but also as a local paracrine factor capable of directly shaping tumour metabolism and cellular identity within CNS malignancies.

### 4.4. Antipsychotic Drug Repurposing as an Anticancer Strategy

Given this biological dependency, pharmacological blockade of DRD2 using established antipsychotic drugs presents a compelling therapeutic strategy. A seminal study by Sachlos and colleagues demonstrated this potential by showing that thioridazine, a first-generation phenothiazine antipsychotic, selectively targets tumour-propagating cells in acute myeloid leukaemia while sparing normal haematopoietic stem cells [25]. Subsequent studies expanded these findings to solid tumours, demonstrating that dopamine receptor antagonists can inhibit proliferation, induce apoptosis, impair self-renewal capacity, and reduce tumour initiation in breast, lung, colorectal, and brain cancer models. To improve translational clarity, the evidence is summarised separately for brain tumour contexts (Table 1) and non-brain tumour contexts (Table 2), including available information on blood–brain barrier penetration, clinical relevance, and mechanistic caveats.

### 4.5. Polypharmacology and Mitochondrial Disruption by Antipsychotics

The antitumour efficacy of these compounds is unlikely to be explained solely by DRD2 antagonism. Many antipsychotic agents possess broad polypharmacologic properties that independently disrupt multiple cellular processes critical for tumour survival [103,104,105]. Phenothiazines, such as chlorpromazine and trifluoperazine, have been shown to impair mitochondrial OXPHOS, alter mitochondrial membrane potential, and increase ROS generation, thereby inducing bioenergetic stress in metabolically active tumour cells, including DMG-relevant models [90,94]. Other studies have demonstrated disruption of autophagic flux, lysosomal function, intracellular calcium homeostasis, and endoplasmic reticulum stress responses following antipsychotic exposure. Trifluoperazine, for example, inhibits calmodulin-dependent signalling and perturbs calcium-mediated survival pathways, whereas chlorpromazine interferes with clathrin-mediated endocytosis and membrane trafficking [91,95]. Collectively, these effects converge on cellular stress pathways that are particularly important for tumour cells with high metabolic demands and limited adaptive reserve.

### 4.6. ONC201/Dordaviprone, Next-Generation Imipridones, and the Convergence of Dopamine Signalling with Mitochondrial Stress

The growing interest in dopamine signalling as a therapeutic target in DMG has been further amplified by the development of ONC201, now also known as dordaviprone, and related imipridone compounds. ONC201 was initially characterised through its ability to antagonise DRD2/3 receptors implicated in tumour cell proliferation, survival signalling, and maintenance of stem-like phenotypes across multiple cancers, including gliomas. More recent pooled clinical data showed single-agent activity of dordaviprone in recurrent H3 K27M-mutant diffuse glioma [79]. In August 2025, the U.S. Food and Drug Administration granted accelerated approval to dordaviprone for adult and paediatric patients 1 year of age and older with H3 K27M-mutant DMG and progressive disease following prior therapy [83]. This approval establishes the clinical relevance of imipridones, while the ongoing ACTION phase 3 trial is testing dordaviprone after frontline radiotherapy in newly diagnosed H3 K27M-mutant diffuse glioma [84].

Mechanistically, the strongest established evidence indicates that ONC201/dordaviprone activates the mitochondrial protease ClpP, leading to disruption of mitochondrial proteostasis, degradation of respiratory chain components, suppression of OXPHOS, mitochondrial damage, and ISR activation characterised by ATF4 and CHOP upregulation [80,81,82]. The contribution of direct DRD2/3 antagonism may vary across tumour models and remains less clearly separable from ClpP-mediated mitochondrial effects. Therefore, in DMG, ONC201 should be considered a dual-activity compound whose therapeutic effects likely reflect convergence between receptor signalling, mitochondrial proteotoxic stress, redox perturbation, and adaptive stress-response failure.

More potent imipridone analogues further support this therapeutic class. ONC206, a fluorinated ONC201 derivative, has demonstrated greater potency than ONC201 in DMG models, stronger ClpP engagement, mitochondrial damage, ROS generation, ISR activation, and antitumour effects in preclinical systems [82,85]. ONC212 and additional analogues also exhibit increased potency in several cancer models and may activate ClpP with distinct pharmacological profiles [80,82]. These compounds are important to mention because the dopamine–mitochondria axis should not be viewed as an ONC201-only concept. However, their clinical relevance in DMG remains investigational, and direct comparisons of efficacy, CNS exposure, tolerability, and resistance mechanisms are still needed.

### 4.7. Current Challenges and Future Directions

Despite these promising findings, substantial translational challenges remain. Antipsychotic drugs are associated with dose-limiting toxicities, including sedation, extrapyramidal symptoms, metabolic dysregulation, and QT interval prolongation, which may restrict their use at concentrations necessary for antitumour activity [106,107]. Furthermore, the broad pharmacological profiles of these agents complicate mechanistic interpretation and biomarker development, making it difficult to distinguish dopamine receptor-dependent effects from off-target metabolic or mitochondrial perturbations. Tumour heterogeneity also represents a major obstacle, as not all cancers exhibit equivalent dependence on DRD2 signalling or mitochondrial stress-response pathways. Consequently, future studies will need to identify predictive biomarkers capable of defining therapeutically vulnerable subgroups and to determine whether dopamine receptor antagonists are best deployed as monotherapies or in rational combination with radiation, epigenetic therapies, or metabolic inhibitors.

Collectively, these findings position dopamine signalling at the intersection of neurotransmitter biology, metabolic regulation, and tumour cell plasticity. The repurposing of antipsychotic agents offers several practical advantages, including established pharmacokinetic profiles, known safety data, and the ability of many compounds to penetrate the blood–brain barrier. Importantly, the convergence of dopamine receptor signalling with mitochondrial stress pathways provides a particularly attractive therapeutic framework in DMG, where epigenetic dysregulation and metabolic dependency are tightly intertwined. Continued mechanistic investigation into how dopaminergic signalling interfaces with mitochondrial function, chromatin regulation, and the tumour microenvironment may therefore reveal novel therapeutic vulnerabilities for aggressive CNS malignancies.

## 5. Dopamine Signalling as a Proposed Metabolic Rheostat: Evidence Base and Mechanistic Model

The dopamine rheostat should be interpreted as a conceptual synthesis rather than a proven linear pathway. Established evidence supports several components of the model: H3K27M reshapes chromatin and developmental cell states; DMG models can display mitochondrial and oxidative stress vulnerabilities; DRD2 signalling regulates metabolic plasticity and pseudohypoxic programmes in glioblastoma stem-like cells; and imipridones can induce ClpP-dependent mitochondrial stress and ISR activation [14,15,18,26,80,81,82,85]. The least established component is the direct claim that endogenous dopamine signalling maintains mitochondrial homeostasis specifically in H3K27M-mutant DMG. This link requires dedicated genetic, pharmacologic, and metabolic validation in patient-derived DMG models.

A plausible mechanistic link between dopamine signalling and mitochondrial homeostasis is mediated through G protein-coupled receptor signalling pathways that interface with metabolic sensors. DRD2 signalling typically inhibits adenylyl cyclase, modulating intracellular cAMP levels and associated calcium flux [108]. In principle, fluctuations in these second messengers could influence rate-limiting TCA cycle enzymes and mitochondrial substrate use, thereby regulating metabolite supply for bioenergetics and epigenetic maintenance [109,110,111]. DRD2 activation can also coordinate PI3K-AKT, MAPK-ERK, and mTOR pathways, which act as metabolic command centres aligning nutrient availability with mitochondrial respiration [26,97,112,113]. However, whether these signalling events operate in the same way in H3K27M-mutant DMG remains to be directly tested.

**Figure 1:**
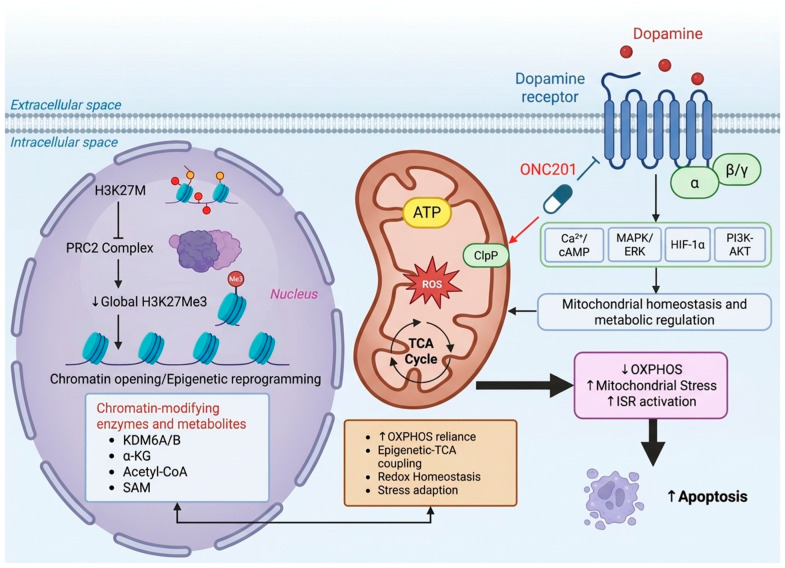
Proposed dopamine–mitochondria–epigenome rheostat in H3K27M-mutant DMG. H3K27M-mediated PRC2 disruption and altered H3K27me3 states reshape chromatin and create metabolic requirements for TCA-derived metabolites, such as alpha-ketoglutarate and acetyl-CoA. Dopamine signalling through DRD2/3 GPCRs at the plasma membrane may modulate cAMP/Ca^2+^, PI3K-AKT, MAPK-ERK, and HIF-1alpha signalling, thereby influencing mitochondrial output, OXPHOS, redox buffering and stress adaptation. ONC201/dordaviprone may disrupt this circuitry through DRD2/3 antagonism and mitochondrial ClpP activation, leading to OXPHOS suppression, ROS accumulation, ISR/ATF4-CHOP activation and apoptosis. The model remains hypothetical and requires direct validation in H3K27M-mutant DMG models. H3K27M, lysine-to-methionine substitution at position 27 of histone H3; PRC2, Polycomb repressive complex 2; H3K27me3, histone H3 lysine 27 trimethylation; Me3, trimethyl group; KDM6A/B, lysine demethylase 6A/B; α-KG, alpha-ketoglutarate; SAM, S-adenosylmethionine; TCA, tricarboxylic acid; ATP, adenosine triphosphate; ROS, reactive oxygen species; ClpP, caseinolytic mitochondrial matrix peptidase proteolytic subunit; ONC201, dordaviprone; Ca^2+^, calcium ion; cAMP, cyclic adenosine monophosphate; MAPK/ERK, mitogen-activated protein kinase/extracellular signal-regulated kinase; HIF-1α, hypoxia-inducible factor 1-alpha; PI3K-AKT, phosphoinositide 3-kinase/protein kinase B; OXPHOS, oxidative phosphorylation; ISR, integrated stress response.

In the context of H3K27M-mutant DMG, we hypothesise that dopamine signalling may function as a metabolic rheostat, a dynamic regulatory system that fine-tunes mitochondrial output and stress-buffering capacity (Figure 1). H3K27M-associated lineage states, including oligodendrocyte progenitor-like and astrocytic-like states, may create heightened dependence on mitochondrial metabolites, such as alpha-ketoglutarate and acetyl-CoA, to sustain the aberrant chromatin landscape [14,15,18]. If dopamine receptor signalling modulates mitochondrial substrate use, calcium handling, redox buffering, or ISR thresholds in these cells, it could help maintain ROS levels below the threshold that triggers cell death. This proposed buffering capacity would be particularly relevant in the metabolically constrained midline microenvironment, but it should be viewed as a testable hypothesis rather than a demonstrated mechanism.

Under this model, imipridones may induce a metabolic catastrophe by disrupting complementary survival mechanisms. ClpP activation directly damages mitochondrial proteostasis and respiratory chain integrity, while DRD2/3 antagonism may remove receptor-mediated survival or stress-adaptation signals in susceptible contexts. The resulting mitochondrial dysfunction, ROS accumulation, and ISR activation can exceed tumour cell buffering capacity and push cells toward apoptosis. Future studies should test this model by separating DRD2-dependent from ClpP-dependent effects using receptor knockout, ClpP loss-of-function, metabolic flux analysis, mitochondrial imaging, and orthotopic DMG models.

## 6. Conclusions

DMG remains one of the most therapeutically challenging paediatric cancers. The discovery of the H3K27M mutation has revealed the central role of epigenetic dysregulation in DMG development, yet effective targeted therapies remain limited. Emerging evidence suggests that epigenetic reprogramming in these tumours may impose metabolic constraints that increase reliance on mitochondrial metabolism and stress-buffering pathways in selected cellular states. Within this context, dopamine receptor signalling may function as a regulatory system that helps stabilise mitochondrial homeostasis through effects on metabolic flux, redox balance, and cellular stress thresholds, but this hypothesis remains to be proven directly in DMG. Future studies integrating metabolic profiling, functional genomics, orthotopic modelling, and pharmacological separation of DRD2/3 and ClpP effects will be essential to determine whether the dopamine–mitochondria axis can be exploited to improve outcomes for patients with this devastating disease.

## Figures and Tables

**Table 1:** Brain tumour-relevant dopamine receptor antagonists and imipridones: mechanisms, CNS penetration, and translational relevance to DMG.

Drug/Class	Brain Tumour Context and Evidence	Mechanistic Relevance	BBB/CNS Penetration and Clinical Relevance
ONC201/dordaviprone	H3K27M-mutant DMG; recurrent/progressive diffuse glioma; early clinical activity and pooled recurrent-disease data [29,30,78,79].	DRD2/3 antagonism was initially emphasised; ClpP agonism, mitochondrial proteostasis disruption, OXPHOS suppression, ISR activation, ATF4/CHOP signalling, and apoptosis are now central mechanisms [79,80,81,82].	Oral CNS-penetrant imipridone; received FDA accelerated approval in 2025 for adult and paediatric patients ≥ 1 year with H3 K27M-mutant DMG with progressive disease after prior therapy [83]. ACTION phase 3 is testing post-radiotherapy dordaviprone in newly diagnosed H3 K27M-mutant diffuse glioma [84].
ONC206	DMG preclinical models; derivative designed to improve potency; in vitro and in vivo DMG studies show bioenergetic disruption and lineage-state effects [85].	Higher ClpP binding affinity and stronger mitochondrial/ISR effects than ONC201 in DMG models; induces mitochondrial damage, ROS, ISR, and apoptosis [82,85].	Brain-tumour relevance is strong but remains investigational; supports reviewer-noted point that more potent imipridone analogues exist [82,85].
Trifluoperazine	Glioblastoma and DMG-relevant preclinical work; reported radiosensitisation and inhibition of mitochondrial function [86,87,88,89,90].	DRD2 antagonism, calmodulin inhibition, impaired DNA repair/autophagy, altered calcium signalling, mitochondrial inhibition, and ROS generation [86,87,90,91].	Clinically used CNS-active antipsychotic with BBB penetration; antitumour concentrations, neurotoxicity, QT risk, and paediatric tolerability limit direct DMG translation.
Chlorpromazine	Glioblastoma models and historical oncology observations [92,93,94].	Bioenergetic stress, interference with pyruvate kinase M2, DNA repair effects, membrane trafficking effects, and chemotherapy/radiotherapy sensitisation [92,93,94,95].	BBB-penetrant antipsychotic; potential repurposing value but sedation, extrapyramidal effects, QT prolongation, and non-selective pharmacology are important translational barriers.
Thioridazine	Glioblastoma and cancer stem cell contexts [25,96].	Cancer stem cell targeting, ER stress, autophagy disruption, DRD2-related effects, and broad phenothiazine polypharmacology [97].	CNS-active but clinical use is limited by cardiotoxicity/QT risk; more useful as proof-of-concept than a near-term DMG therapy.
Haloperidol/aripiprazole	Glioblastoma/glioma preclinical studies [76,98].	DRD2 pathway modulation; haloperidol has been linked to ER stress, ferroptosis/autophagy, and temozolomide sensitisation; aripiprazole has been linked to Src signalling [76,98].	BBB-penetrant clinical antipsychotics; DMG-specific evidence is limited and requires direct testing.

**Table 2:** Non-brain tumour evidence for repurposed antipsychotics and imipridone analogues relevant to dopamine–mitochondrial stress biology.

Drug/Class	Non-Brain Tumour Contexts	Main Reported Anticancer Mechanisms	Translational Caveats/ Relevance to DMG
Trifluoperazine	Colorectal, breast, and lung cancer models [87,88,89,99].	G0/G1 arrest, apoptosis, cancer stem-like cell targeting, EGFR inhibitor/chemotherapy resistance reversal, calmodulin-dependent signalling disruption [87,88,89,91,99].	Supports broad anticancer activity of DRD2-active phenothiazines, but mechanisms are highly pleiotropic and cannot be attributed solely to DRD2 blockade.
Pimozide	Breast and lung cancer models [100].	Cell-cycle arrest, DNA double-strand breaks, apoptosis, anti-angiogenic and stromal effects [100].	CNS penetration is pharmacologically established, but DMG-specific evidence is lacking; QT prolongation risk is relevant.
Penfluridol	Renal cell carcinoma, prostate, and breast cancer models [101,102].	UPR signalling, autophagy-mediated apoptosis, GLI1/OCT4/Nanog suppression, stemness inhibition, and synergy with sunitinib [101,102].	Long-acting antipsychotic with CNS activity; limited DMG evidence and potential toxicity require caution.
Chlorpromazine	General oncology and schizophrenia-cohort observations [93,94].	Radiation/chemotherapy sensitisation, mitochondrial bioenergetic disruption, endocytosis and membrane-trafficking effects [93,94,95].	Useful mechanistic comparator for mitochondrial and stress-pathway disruption, but clinical translation to DMG would require dose and safety optimisation.
Thioridazine	Acute myeloid leukaemia and solid-tumour cancer stem cell models [25].	Preferential targeting of tumour-propagating cells; DRD2-related differentiation and survival effects [25].	Illustrates dopamine receptor antagonist repurposing; cardiotoxicity limits direct therapeutic repositioning.
ONC212 and other imipridone analogues	Multiple solid and haematologic cancer models [81,82].	Potent ClpP activation, OXPHOS suppression, mitochondrial proteotoxic stress, ISR activation, and apoptosis; ONC212 may also engage GPR132/Galphaq signalling [81,82].	Demonstrates that imipridones extend beyond ONC201 and may offer higher potency; CNS and DMG-specific therapeutic windows require further validation.

## Data Availability

No new data were created or analysed in this review. Data sharing is not applicable.
